# Microenvironmental derived factors modulating dendritic cell function and vaccine efficacy: the effect of prostanoid receptor and nuclear receptor ligands

**DOI:** 10.1007/s00262-018-2205-1

**Published:** 2018-07-11

**Authors:** Tonke K. Raaijmakers, Marleen Ansems

**Affiliations:** 1 Radiotherapy and OncoImmunology Laboratory, Department of Radiation Oncology, Radboud Institute for Molecular Life Sciences, Radboud University Medical Center, Geert Grooteplein Zuid 32, 6525 GA Nijmegen, The Netherlands; 2 Department of Anesthesiology, Pain and Palliative Medicine, Radboud University Medical Center, Geert Grooteplein 10, 6525 GA Nijmegen, The Netherlands

**Keywords:** Dendritic cells, Vaccine, Immunotherapy, Microenvironment, Nuclear receptors, Prostanoid receptors

## Abstract

Dendritic cells (DCs) are widely used in DC-based immunotherapies because of their capacity to steer immune responses. So far treatment success is limited and more functional knowledge on how DCs initiate and stably drive specific responses is needed. Many intrinsic and extrinsic factors contribute to how DCs skew the immune response towards immunity or tolerance. The origin and type of DC, its maturation status, but also factors they encounter in the in vitro or in vivo microenvironment they reside in during differentiation and maturation affect this balance. Treatment success of DC vaccines will, therefore, also depend on the presence of these factors during the process of vaccination. Identification and further knowledge of natural and pharmacological compounds that modulate DC differentiation and function towards a specific response may help to improve current DC-based immunotherapies. This review focuses on factors that could improve the efficacy of DC vaccines in (pre-)clinical studies to enhance DC-based immunotherapy, with a particular emphasis on compounds acting on prostanoid or nuclear receptor families.

## Introduction

Dendritic cells are essential mediators of immunity and tolerance as they play a crucial role in the initiation and modulation of the immune response. They recognize and take up antigens in the periphery and upon maturation migrate to the lymph nodes and present the antigens to naïve T cells. DCs are very plastic and have the ability to adapt to the microenvironment they reside in via modulation of their phenotype and function. The heterogeneity among DCs is of particular interest due to the specialized functional property of each DC subset. DCs are classically divided into multiple subsets, including plasmacytoid DCs and two functionally specialized subsets of conventional DCs (cDCs), termed cDC1 and cDC2 [1, 2].

Because of their important role in initiating and skewing particular immune responses, DCs are widely used in preclinical mouse models as well as clinical studies to boost anti-tumor immunity, to treat autoimmune diseases or to prolong graft survival in transplantation. Despite the emergence of anti-cancer immunotherapy with immune checkpoint blockade, currently still many clinical trials involving DC vaccines are ongoing [3] showing that the field of DC-based cancer immunotherapy is still very active. Importantly, DC-vaccines are well tolerated and often anti-tumor immune responses with clinical benefit are generated, however, durable responses and long-term survival effects in cancer patients are less clear. So far, none of the current single treatment modalities have shown effectiveness in all patients. Therefore, also many DC-based immunotherapies are used in combination with other therapies including immune checkpoint blockade, adoptive T cell therapy, chemotherapy and radiation therapy to improve long-term survival of cancer patients [4–6].

**Fig. 1:**
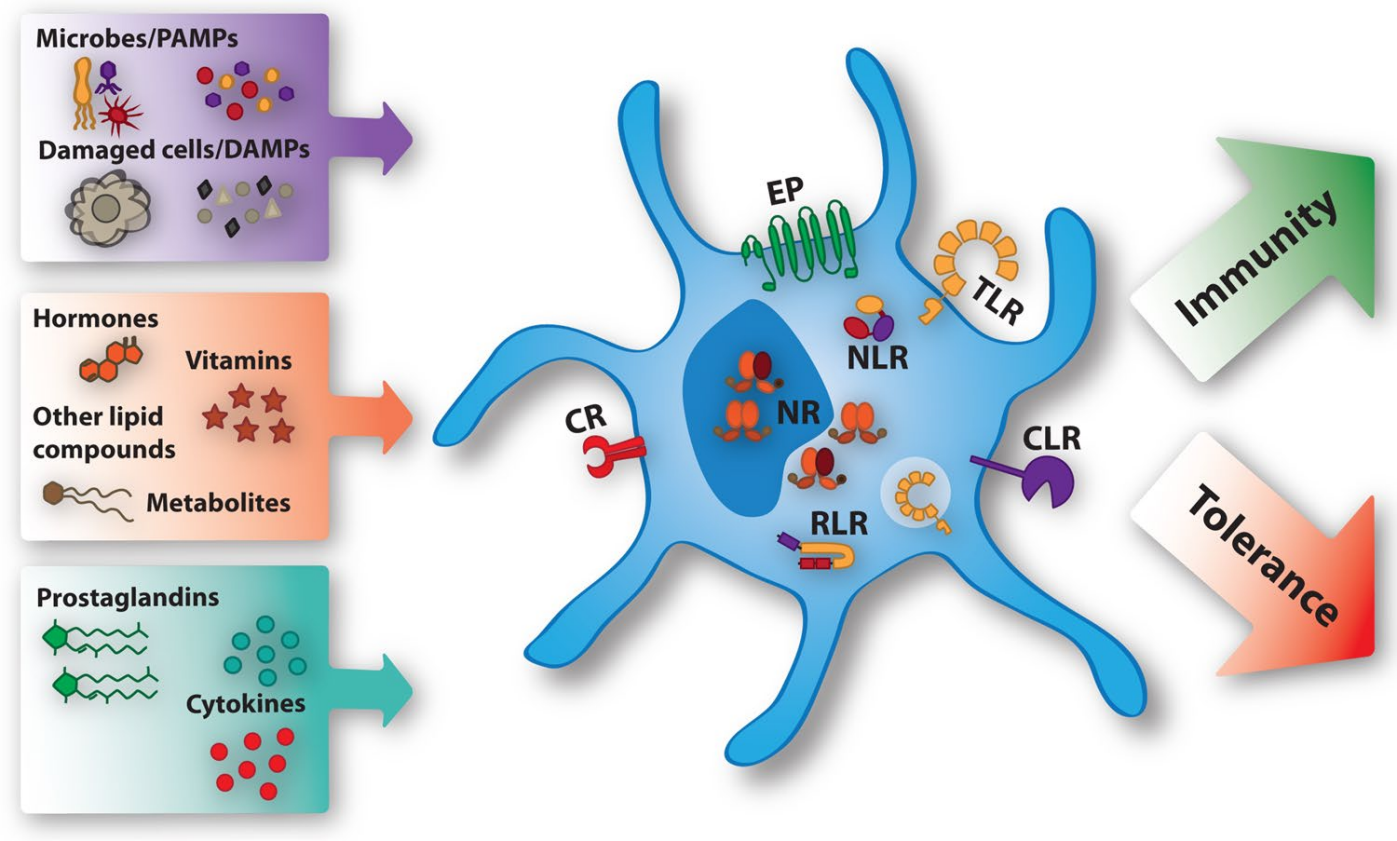
Ligands modulating DC function via specific receptors. The microenvironment contains many ligands that differentiate DCs more towards an immune promoting or immunosuppressive phenotype. PAMPs and DAMPs bind to extracellular and intracellular Toll-like receptors (TLRs), C-type lectin receptors (CLRs), NOD-like receptors (NLR), and Rig-I-like receptors (RLRs). The effects of the prostaglandins are mediated by E-type prostanoid receptors (EP). Cytokines are recognized by specific cytokine receptors (CR). Hormones, vitamins, other lipid compounds, and metabolites exert their function through binding specific nuclear receptors (NR) present in the nucleus or the cytoplasm

The efficacy of DC-based therapies can be influenced by many different factors, including the maturation status of the DC, the nature, source and delivery strategy of tumor-associated antigens to the DC, the dose and frequency of the vaccine, the adjuvants used, the route of administration as well as the DC subset that was targeted or used. Strategies that have been proposed and performed to improve the efficacy of DC-based immunotherapy, include the induction of immunogenic cell death, interfering with immunosuppressive networks, overcoming metabolic constraints and modulating the microbiome (reviewed in [7]). The success of all these strategies depends on how a particular DC responds to the myriad of factors it encounters during its generation, differentiation, the process of maturation and migration to the lymph node. Via distinct receptors DCs recognize, adapt and respond to the different factors present in the microenvironment (see Fig. 1). These factors include cytokines, pathogen-associated molecular patterns (PAMPs), endogenous danger-associated molecular patterns (DAMPs), prostaglandins, hormones, vitamins and other lipid compounds and metabolites. Conserved structures derived from microorganisms and damaged cells, the PAMPs and DAMPs, are recognized via Pattern recognition receptors (PRRs). Distinct classes of PRRs exist, one of which are the TLRs. TLRs are transmembrane receptors that are expressed on the plasma membrane or the endosomal compartment of the cell. They recognize a variety of conserved microbial structures, such as RNA, DNA and peptides. Another class of PRRs are the C-type lectin receptors (CLRs), which recognize conserved carbohydrate residues. In contrast to the membrane bound TLRs and CLRs, the nucleotide-binding oligomerization domain (NOD)-like receptors (NLRs) and retinoic acid-inducible gene (RIG)-I-like receptors (RLRs) are cytoplasmic PRRs. The NLRs are involved in sensing the presence of intracellular microorganisms and the last class of PRRs, the RLRs sense intracellular viral replication through binding of viral nucleic acids. Prostaglandins are specifically recognized by E-type prostanoid receptors (EP), while many hormones, vitamins, lipid compounds and metabolites are recognized by different types of nuclear receptors (NRs). Natural and pharmacological compounds triggering these receptors have been used extensively to modify and enhance the efficacy of DC vaccines in different settings. This review focuses on the factors modulating DC function that signal via prostanoid receptors and NRs (see Table 1).

**Table 1:** Summary table of the different modes of action of prostanoid and nuclear receptors and their ligands in DCs

Receptor	Ligand	Immunogenic properties	Tolerogenic properties
EP1-4	PGE2	Induction of CD80, CD86, CCR7 [18] and MMP-9 [17]	Inhibition of IL-12p70 [22] and chemokine receptor expression [26], induction of IL-10 [15, 23]
RAR/RXR	Retinoids		Inhibition of IL-12 and increase of IL-10 [45], production of RA leading to the attraction of regulatory T cells [43, 44]
GR	Corticosteroids		Inhibits maturation and pro-inflammatory cytokine production, strong induction of IL-10 [50]
VDR	VDR ligands		Inhibits differentiation and maturation and stimulates Treg induction [39, 52]
PPARγ	PPARγ ligands	Induction of Th2 immunity [59]	Induction of regulatory mucosal phenotype [57]
LXR	LXR ligands	Increases maturation [63], required for DC-migration in response to CCR7 ligands [65]	Inhibits maturation [61, 62], inhibits CCR7-dependent migration [64]
Nurr1			Orchestrates expression of immunoregulatory genes [74]
NOR-1		Required for TLR-mediated DC maturation [76] Required for CCR7 dependent DC migration [73]	

## Prostanoid receptor ligands

Prostaglandins are small, physiologically active lipid compounds with very diverse effects in the body, affecting kidney function, platelet aggregation, neurotransmitter release, and modulation of immune cell function, including DCs [8]. They have been shown to exhibit a wide range of effects on DC function such as maturation, cytokine excretion, homing and T cell activation [9]. Prostaglandins are metabolized by two COX enzymes from arachidonic acid, released from lipid membranes [8–11], including lipid droplets [12]. COX1 is constitutively expressed at low levels in most tissues, and maintains homeostatic levels to regulate normal physiological function, whereas COX2 is generally undetectable, but induced in response to inflammation and stress stimuli, and therefore, responsible for large fluctuations in prostaglandin levels [8]. One of the best-studied and most abundant prostaglandin in inflammatory milieus is prostaglandin E2 (PGE2). It mediates pyrexia, hyperalgesia, arterial dilatation, and is a potent modulator of the immune system including DCs [8, 9].

Prostaglandins act in an autocrine and paracrine fashion via four distinct E-type prostanoid receptors (EP) termed EP1-4 [10, 13]. EP1-4 are rhodopsin type, G protein-coupled receptors [8, 10, 13]. They are associated with different G proteins, thereby inducing different second messenger signaling pathways, modulating the diverse functions of the prostaglandins [8–10, 13]. The receptors are generally believed to be expressed on the plasma membrane [10, 11, 13]. However, some reports note additional subcellular distributions, within and around the nucleus [8, 10]. Depending on the DC subset studied, it was found that DCs express either EP2 and EP4 [14], EP2, EP3 and EP4 [15] or all four EP receptors [11, 16]. However, the use of selective inhibitors have shown that PGE2 mainly exerts its effect via EP2 and EP4 on human moDCs [14].

Prostaglandins, produced by DCs themselves or by surrounding cells, can have stimulating as well as inhibiting effects on DCs, depending on the site of encounter, the maturation stage of the DC, the concentration and the prostanoid receptors activated. In the periphery, PGE2 drives pro-inflammatory responses in immature DCs, by inducing DC activation and migration. The expression of the maturation markers CD80 and CD86 increases upon PGE2 exposure [17, 18]. In addition PGE2 increases CCR7 [17, 18] and MMP-9 [17] expression, both of which are necessary for the migration of DCs toward the lymph node derived chemokines CCL19 and CCL21. PGE2-mediated migration and maturation of Langerhans cells were also confirmed in vivo, and are mediated via EP4 [19]. When DCs are matured with IFNɑ and TNFɑ, however, additional PGE2 exposure lowers CD40 and CD86 expression [17], indicating an immunosuppressive effect of PGE2 on these mature DCs. Furthermore, PGE2 has T cell priming [20] as well as T cell polarizing effects via affecting cytokine secretion by DCs [21]. The production of Th1-inducing cytokine IL-12p70 is inhibited by PGE2 in DCs stimulated with the TLR4 ligand LPS [22], whereas PGE2 stimulates the production of the immune suppressive cytokine IL-10 [15, 23] by immature DCs. Overall, PGE2 is considered to bias the immune response away from Th1 responses towards Th2 [8, 22]. Interestingly, PGE2 can also affect IL-23 production by DCs and thereby Th17 differentiation [24, 25]. PGE2 mediated regulation of IL-23 expression in DCs is concentration dependent and affected by the specific EP activated. EP4 stimulation with low PGE2 concentrations leads to high IL-23 production, whereas EP2 stimulation with high PGE2 concentrations results in lower IL-23 production [15]. Furthermore, activation of EP2 and EP4 with PGE2 results in a decreased ability of mouse bone marrow derived DCs to induce proliferation of allogeneic T cells [11].

Also tumor-derived PGE2 has been described to profoundly affect the function of DCs, as it regulates the interplay between NK cells and cDC1s in the tumor microenvironment, cells that are both critical in generating an antitumor response [26]. PGE2 reduced the production of DC-recruiting chemokines by NK cells, partly via reduced NK cell survival. This inhibited the migratory responsiveness of cDC1s to these chemokines, which is important for the recruitment of these cells towards the tumor microenvironment and ultimately for inducing an effective antitumor immune response [26].

The maturation status, migratory potential, and cytokine production are all important features of DCs that need to be optimal for DC-vaccines to be effective [27]. Current DC vaccination strategies as anti-cancer therapy involve *ex vivo* maturation and loading with antigens of the DCs [27, 28]. Monocyte derived autologous DCs, currently the most frequently used for DC-based immunotherapies, are differentiated from patients own peripheral blood monocytes and loaded with tumor antigens *ex vivo*. In initial clinical studies, *ex vivo* maturated DCs were relatively immature [27] and were shown to have limited migratory capacity and T cell activating potential. To increase the migratory capacity PGE2 was included in the maturation cocktail [14]. Nowadays, the maturation cytokine cocktails used in the literature are diverse [29], but the golden standard cocktail for ‘second-generation’ DC-based vaccines includes TNFα, IL-1β, IL-6, and PGE2 [30]. This maturation cocktail effectively matures DCs, and enables them to migrate from the periphery towards the lymph nodes. Indeed, PGE2-matured DCs migrate more efficiently to lymph nodes than immature DCs, however, it is not critically required [9]. Importantly, as DCs matured in the presence of PGE2 also show an exhausted phenotype [9, 27], with a reduced ability to produce the Th1 steering cytokine IL-12p70, the optimal combination for *ex vivo* DC maturation, without PGE2, is also explored [9, 27, 30]. Replacing PGE2 and IL-6 with IFNα, IFNγ and poly-I:C in the maturation cocktail resulted in “α-type-1-polarized DCs” which are non-exhausted and lead to a better potential to generate tumor-specific CD8 + T cells [31]. Another strategy is the use of naturally occurring DC subsets, including primary myeloid DCs [32] and plasmacytoid DCs [33] that do not need PGE2 to obtain their full potential and create a favorable immune response.

## Nuclear receptors and their ligands

Besides prostanoid receptor ligands, also NR ligands are known to play an immune modulatory role in DCs. NR ligands are hydrophobic derivatives of retinoids, lipophilic hormones and vitamins, cholesterol, xenobiotics and synthetic drugs [34]. NR ligands bind to NRs, thereby modulating their activity. NRs are transcription factors that regulate gene expression and affect various processes such as homeostasis, reproduction, embryonic development, cell differentiation, but also the immune response [35]. The NR superfamily in humans consists of 48 members that are generally divided in three main classes. The first class, the steroid receptors, consists of cytosolic NRs that after binding to a ligand dimerize to form homodimers and translocate to the nucleus. In the nucleus, the NR homodimer binds to hormone response elements in the DNA to modulate transcription of target genes. The second class of NRs, the retinoid X receptor (RXR) heterodimers, are localized in the nucleus and bind to response elements in the DNA as heterodimers with RXR. Upon ligand binding the heterodimers modulate target gene expression. The third class of NRs are the orphan receptors, which either have a yet unidentified ligand or do not require a ligand to function. Many efforts are ongoing in finding natural or pharmacological agents targeting the orphan receptors [36]. All NR family members share a similar structure, containing an amino terminal activation domain, a DNA-binding domain, a ligand-binding domain, and a second carboxy terminal activation domain. NRs recognize and bind to specific DNA response elements in genes and undergo conformational changes resulting in the recruitment or release of co-repressors or co-activators, leading to inhibition or initiation of transcription of the gene [37]. Many members of the NR superfamily, but also NR-ligand-metabolizing enzymes are expressed by DCs [38–40]. We have recently shown that different murine DC subsets show the same repertoire of NRs, although the expression levels vary [38]. The distinct DC subsets may, therefore, react differently to NR ligands present in unique microenvironments. Certain NRs have clear effects on DC function, therefore, multiple (pre-)clinical studies have used natural and pharmacological compounds to activate or repress these NRs to improve DC-based immunotherapy. Below we will describe the effects of the NRs most well known to affect DC function: the steroid receptor: glucocorticoid receptor (GR), the RXR heterodimers: retinoic acid receptor (RAR), vitamin D receptor (VDR), peroxisome proliferator-activated receptor γ (PPARγ), liver X receptor (LXR) and the orphan NRs: Nur77, Nurr1 and NOR-1 which belong to the nuclear receptor 4A (NR4A) subgroup of NRs.

### RAR/RXR

Vitamin A and its derivatives retinoids are ligands for RAR and RXR. Multiple reports have shown that development of mucosal DCs as well as optimal DC function in the microenvironment of the gut and in the mesenteric lymph nodes are dependent on the vitamin A derivative retinoic acid (RA) [41, 42]. RA conditioned DCs express the metabolizing enzyme retinal aldehyde dehydrogenase, which enables them to produce RA themselves, important for the attraction of gut homing regulatory T cells [43, 44]. Overall, DCs exposed to RA have a more tolerogenic phenotype, with decreased production of IL-12 and increased production of IL-10 [45]. To boost DC function, preclinical studies have, therefore, used a RARα antagonist in an antigen-pulsed and TLR-activated DC vaccine against B16 melanoma and showed that inhibition of RA indeed enhances the efficacy of the DC vaccine [46]. In addition to its effects on DCs, retinoids are also known to enhance the pro-apoptotic effects of type I IFN in tumor cells [47]. In this light, RA together with IFNα has been used to enhance the induction of immunogenic cell death of tumor cells. This allowed the generation of a highly immunogenic antigen source that improved the therapeutic potential of DC-based immunotherapy in a preclinical mouse model of lymphoma [48].

### GR and VDR

Ligands for both GR and VDR are linked to immune suppression, and therefore, widely used as immunosuppressive drugs for the treatment of multiple autoimmune diseases. Also in DCs, we and others have shown that the use of agonists for both receptors is associated with the generation of tolerogenic DCs [39, 49]. Corticosteroids, including dexamethasone as agonist for GR, inhibit DC maturation by repression of the production of pro-inflammatory cytokines and strong induction of the anti-inflammatory cytokine IL-10 [50], which is partly mediated by the expression of the GR target gene glucocorticoid-induced leucine zipper (GILZ) [49, 51]. 1,25(OH)2D3, the ligand for VDR, inhibits the differentiation, maturation and immunostimulatory capacity of DCs and is associated with regulatory T cell induction [39, 52]. Additionally DCs themselves can also provide a local source of bioactive ligands for VDR and thereby modulate T cell responses [53]. Because of their strong tolerogenic character, ligands for these NRs are successfully applied to obtain tolerogenic DCs for use in multiple preclinical immunotherapy studies in transplantation medicine, allergy and autoimmunity (reviewed in [54, 55]). In contrast to obtaining tolerogenic DCs using ligands for GR and VDR, blocking gene expression of the GR target gene GILZ boosted DC activation and enhanced the efficacy of DC-vaccines in a mouse model of B-cell lymphoma [56].

### PPARγ and LXR

Although PPARγ activation has mainly been reported to mediate the resolution of inflammation and induce tolerogenic DCs [57, 58], it has also been described that PPARγ can have a proinflammatory role in DCs in type-2 immunity [59]. This effect is thought to be dependent on the given tissue context and additional signs like PAMPs and cytokines. In lung-resident CD11b + DCs, PPARγ promotes the induction of Th2 immunity [59], whereas PPARγ activation in bone marrow derived DCs induces a more regulatory mucosal phenotype [57]. Similar to PPARγ activation, also LXR activation has shown contradictory results regarding its effect on DCs. Next to its effect on DC differentiation [60], it has been shown to inhibit DC maturation [61, 62] as well as sensitize DCs to inflammatory stimuli increasing DC maturation [63]. Also opposing effects of LXR activation on CCR7 dependent migration have been reported. While activation of LXR has been shown to inhibit CCR7-dependent DC migration to secondary lymph nodes [64], it has also been shown to be required for DC migration in response to CCR7 ligands [65]. Interestingly, PGE2 has been shown to counterbalance LXR-dependent dampening of CCR7 expression and DC migration, independent of prior LXR activation [66], showing the complexity of how DCs can be regulated by different lipid derivatives.

Modulating the function of both PPARγ and LXR in DCs has already been successfully applied in multiple preclinical immunotherapy studies using DC vaccines. While activating PPARγ with its agonist rosiglitazone was used to generate a tolerogenic DC vaccine to ameliorate collagen-induced arthritis in mice [67], systemically inhibiting LXR activation by blocking cholesterol/oxysterol synthesis with zaragozic acids was used to increase the efficacy of DC vaccination in tumor-bearing mice [68]. However, as interfering with cholesterol synthesis not only inhibits LXR activation, but also affects CCR7-driven DC migration [69], care should be taken. Interestingly, both PPARγ and LXR signalling also inhibit DC-mediated trans-infection of HIV-I to T cells mediated via a decrease in DC-associated cholesterol, required for DC capture of HIV-1 [70]. This capacity also shows the potential therapeutic value of targeting these NRs in DCs in inhibiting HIV-1 mucosal transmission or potentially more general DC-based vaccines stimulating pathogen-specific immune responses.

### NR4A

We and others have recently shown that all three members of the NR4A subgroup of NRs, Nur77 (NR4A1), Nurr1 (NR4A2) and NOR-1 (NR4A3) are expressed in different subsets of DCs [38, 71–74]. NR4A receptors are orphan NRs that have no identified endogenous ligand and function in a ligand-independent manner. Instead, NR4A activity is dependent on expression of the receptor, posttranslational modifications and induction by kinases, phosphatases, hormones, vitamins, and cellular stress [75]. Nurr1 has recently been shown to induce a tolerogenic phenotype in murine bone marrow derived DCs [74], while NOR-1 has been reported to be involved in TLR-mediated DC maturation [76], DC migration [73] and activation-induced cell death in DCs [71], indicating that these receptors could be interesting targets in DC-based immunotherapy. The role of Nur77 in DCs remains more elusive; therefore, current studies of our lab aim to elucidate the role of Nur77 in different subsets of DCs. Silencing NOR-1 in murine DCs has previously been shown to improve the short-term survival efficacy of a DC vaccine in a B-cell lymphoma model in mice [71]. Current efforts are aiming at generating specific compounds that will induce or repress the expression, or alter the posttranslational modification of individual NR4As to specifically modulate DC function.

## Concluding remarks

DC-based immunotherapy has the potential (alone or in combination with other therapies) to make a difference in cancer therapy. Many natural and chemical compounds have successfully been used to improve (pre-)clinical DC-based immunotherapies. However, the multifaceted biology of many of these factors emphasize the importance of ongoing efforts into a deeper understanding on the precise effect of these factors in DC function. PGE2 is currently part of the golden standard cytokine mix to mature DCs ex vivo, however, the immunosuppressive effects of PGE2 on DC function must not be overlooked. Also many NRs have a complex and dual effect on DC function. Considering that DCs encounter many different ligands of these receptors during differentiation and maturation (ex vivo) or after injection (in vivo), we need to keep reflecting on how the presence of these ligands will affect DC-vaccination efficacy. It will be important to find or develop ligands that can specifically activate or repress the function of individual receptors, thereby creating the possibility of perfectly shaping DCs or specific DC subsets for optimal use in DC vaccination strategies.

## Abbreviations

cDC: Conventional dendritic cell
CLR: C-type lectin receptor
DAMP: Damage associated molecular pattern
EP: E-type prostanoid receptor
GR: Glucocorticoid receptor
LXR: Liver X receptor
NLR: NOD-like receptor
NOD: Nucleotide-binding oligomerization domain
NR: Nuclear receptor
NR4A: Nuclear receptor 4A
PAMP: Pathogen associated molecular pattern
PGE2: Prostaglandin E2
PPARγ: Peroxisome proliferator-activated receptor γ
PRR: Pattern recognition receptor
RAR: Retinoic acid receptor
RIG: Retinoic acid-inducible gene
RLR: RIG-I-like receptor
RXR: Retinoid X receptor
VDR: Vitamin D receptor
